# Induction of antigen-specific regulatory T cells by engineered extracellular vesicles

**DOI:** 10.1080/10717544.2025.2586305

**Published:** 2025-12-18

**Authors:** Shota Imai, Kanto Nagamori, Uryo Onishi, Xiabing Lyu, Iriya Fujitsuka, Makie Ueda, Tomoyoshi Yamano, Rikinari Hanayama

**Affiliations:** a Department of Immunology, Graduate School of Medical Sciences, Kanazawa University, Kanazawa, Ishikawa, Japan; b WPI Nano Life Science Institute (NanoLSI), Kanazawa University, Kanazawa, Ishikawa, Japan

**Keywords:** Extracellular vesicles, exosome, regulatory T cells, antigen-specific immunotherapy, TGF-β, immune tolerance

## Abstract

Extracellular vesicles (EVs) are emerging as versatile nanocarriers for targeted drug delivery and immune modulation. However, strategies that can induce antigen-specific immune tolerance remain limited, highlighting an unmet need for more precise and effective approaches. To address this challenge, we aimed to develop a modular EV-based system capable of inducing antigen-specific regulatory T cells (Tregs). In this study, we developed engineered antigen-presenting EVs (AP-EVs) that co-display peptide–major histocompatibility complex class II complexes (pMHCII), interleukin−2 (IL−2), and transforming growth factor-*β* (TGF-*β*) on their surface. These immunomodulatory molecules were anchored to the EV membrane via CD81 or milk fat globule-EGF factor 8 (MFG-E8) scaffolds to ensure stable and multivalent presentation. AP-EVs induced the differentiation of antigen-specific Tregs from naïve CD4⁺ T cells *in vitro*, and promoted their proliferation and expression of canonical regulatory markers, including CD25, CTLA−4, PD-L1, and LAG−3. *In vivo*, the combination of AP-EVs and mTOR inhibition with rapamycin significantly enhanced the generation of Foxp3⁺ Tregs in antigen-specific adoptive transfer models. The Tregs induced by AP-EVs *in vitro* exhibited suppressive function, highlighting the therapeutic potential of this system. Our findings establish a modular, cell-free EV platform for antigen-specific immune tolerance, with potential applications in the treatment of autoimmune and allergic diseases through targeted immune regulation.

## GRAPHICAL ABSTRACT

**Figure:**
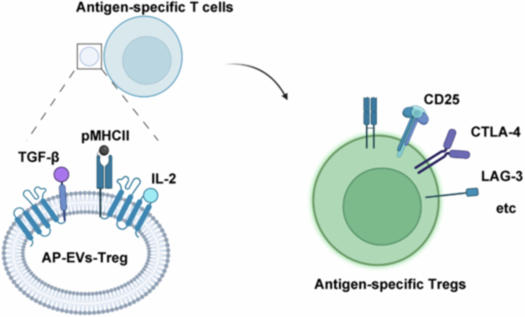

## Introduction

Autoimmune diseases are characterized by an aberrant immune response in which the immune system mistakenly attacks the body's own tissues (Davidson and Diamond 2001; Pisetsky 2023; Song et al. 2024). Current therapies for autoimmune diseases mainly rely on broad immunosuppression, including corticosteroids and biologic agents targeting inflammatory cytokines. While these treatments alleviate symptoms and slow disease progression, they are inherently non-specific and often cause serious side effects such as increased susceptibility to infections and heightened risk of malignancies (Her and Kavanaugh 2016). Moreover, they do not correct the underlying immune dysregulation and rarely achieve long-term remission. Therefore, there is an urgent need for therapeutic strategies that can re-establish immune tolerance in an antigen-specific manner. In this study, we aimed to develop an engineered extracellular vesicle (EV)–based platform capable of delivering peptide–major histocompatibility complex class II complexes (pMHCII) together with immunoregulatory signals to selectively induce antigen-specific regulatory T cells (Tregs).

In recent years, Tregs have emerged as a promising strategy for the treatment of autoimmune diseases (Goswami 2022; Sakaguchi et al. 2023). Tregs are a specialized subset of CD4⁺ T cells that play a crucial role in maintaining immune homeostasis and preventing autoimmunity by suppressing aberrant immune responses. Dysregulation in the number or function of Tregs has been implicated in the pathogenesis of several autoimmune diseases (Sakaguchi et al. 1995; Sakaguchi et al. 2008; Josefowicz et al. 2012). This finding suggests that restoring Treg function or increasing Treg numbers could be a promising therapeutic approach for autoimmune disorders.

One of the most promising approaches involves restoring immune tolerance by harnessing Tregs. Therapeutic strategies aim to either enhance endogenous Treg function or administer *ex vivo* expanded Tregs to patients. While polyclonal Tregs targeting a wide range of antigens have shown some efficacy, they pose risks such as the non-specific suppression of protective immune responses (Qu et al. 2022). To overcome these challenges, the development of antigen-specific Tregs has attracted considerable attention. These Tregs are engineered or selected to target disease-relevant antigens, thereby enabling localized and precise immunosuppression without compromising systemic immunity. This approach not only enhances therapeutic efficacy but also minimizes the side effects associated with broad immunosuppression (Sakaguchi et al. 2023).

A subset of CD4⁺ T cells differentiate into Foxp3-expressing Tregs (thymic Treg, tTreg) within the thymus, where they play a critical role in maintaining central immune tolerance (Sakaguchi et al. 2008). In the periphery, peripheral Tregs (pTreg) are generated from conventional CD4⁺ T cells (Tconv), particularly in the intestinal environment, in response to microbial metabolites or upon exposure to high concentrations of retinoic acid and TGF-*β* (Coombes 2007; Sun 2007). Moreover, induced Tregs (iTreg) can be generated *in vitro* from Tconv cells under defined conditions, such as in the presence of TGF-*β* and IL−2 (Chen 2003; Gu et al. 2022).

A recent study by Krienke demonstrated the feasibility of generating antigen-specific Tregs using mRNA-based technology, showing promising outcomes in mouse models of immune tolerance (Krienke 2021; Wardell and Levings 2021). Despite these promising results, mRNA-based therapies face several limitations. Potential concerns include the immunogenicity of mRNA delivery systems, unintended off-target effects, and the transient nature of mRNA expression, which may necessitate repeated dosing (Pardi et al. 2018).

Here, building on our recent findings (Kimura 2025; Lyu 2025), we introduce an EVs-based platform for the induction of antigen-specific Tregs. EVs, including exosomes, are promising nanoscale carriers for clinical applications due to their excellent biocompatibility, low immunogenicity, and high efficiency in drug delivery (Cheng and Hill 2022). EVs contain a diverse array of proteins, lipids, and RNAs that can induce functional and physiological changes in recipient cells (van Niel et al. 2018). In our previous studies, we developed antigen-presenting extracellular vesicles (AP-EVs) that selectively activated antigen-specific CD8⁺ and CD4⁺ T cells *in vivo* by displaying pMHCII and key immunomodulatory factors. In particular, EVs engineered with MHCI and MHCII, together with cytokines such as IL−2, IL−12, and IL−4, effectively promoted the expansion and differentiation of tumor antigen-specific cytotoxic and helper T cells, respectively. Inspired by these results, we now apply this modular EV platform to induce antigen-specific Tregs by displaying pMHCII together with IL−2 and TGF-*β*, providing a novel, cell-free, and scalable approach for restoring immune tolerance in autoimmune diseases.

## Materials and methods

### Cell lines

Human embryonic kidney (HEK)-293T cells (ATCC, Manassas, VA, USA, Cat# CRL−3216) and Lenti-X 293 T cells (Takara Bio, Shiga, Japan, Cat# 632180) were cultured in Dulbecco’s modified Eagle’s medium (DMEM; Thermo Fisher Scientific, Waltham, MA, USA) supplemented with 10% heat-inactivated fetal calf serum (FCS; Thermo Fisher Scientific, Waltham, MA, USA), 100 U/mL penicillin, and 100 U/mL streptomycin (FUJIFILM Wako, Osaka, Japan). Murine lymphocytes were cultured in RPMI (Nacalai Tesque, Kyoto, Japan) supplemented with 10% FCS, 1 × non-essential amino acid (Nacalai Tesque, Kyoto, Japan), 1 nmol/L sodium pyruvate (Nacalai Tesque, Kyoto, Japan), 100 U/mL penicillin, 100 U/mL streptomycin (FUJIFILM Wako, Osaka, Japan), and 0.05 μM 2-mercaptoethanol (Thermo Fisher Scientific, Waltham, MA, USA). The AP-EV-producing cell line was cultured in FreeStyle™ 293 Expression Medium (Gibco, Waltham, MA, USA) supplemented with 100 U/mL penicillin, and 100 U/mL streptomycin.

### Preparation and purification of AP-EVs

4.0 × 10^6^ HEK293T cells were plated in a 10-cm-dish a day before transfection. Cells were transfected with 20 µg of the plasmids using 80 µg of polyethylenimine (PEI; Polysciences, Warrington, PA, USA). The cell culture media was replaced with FreeStyle™ 293 Expression Medium 8−16 h after transfection, and cells were cultured for another 2 days to produce AP-EVs. For AP-EVs purification, the cell culture supernatant was first centrifuged at 500 × g for 5 min to remove cell debris and then re-centrifuged at 2,000 × g for 20 min to remove cell debris and apoptotic bodies. The supernatant was again centrifuged at 10,000 × g for 30 min to remove apoptotic bodies and large EVs. AP-EVs were isolated from the supernatant through centrifugation at 100,000 × g for 3 h, the pellet was washed with phosphate-buffered saline (PBS; FUJIFILM Wako, Osaka, Japan). AP-EVs concentration was determined using a bicinchoninic acid (BCA) assay (Thermo Fisher Scientific, Waltham, MA, USA) or Nanoparticle tracking analysis.

### Mice

C57BL/6 mice were purchased from Japan SLC (Shizuoka, Japan) and The Jackson Laboratory (Bar Harbor, ME, USA). OT-II TCR transgenic mice (expressing a T cell receptor specific for the Ovalbumin (OVA)_323–339_ peptide presented by I-A^b^) and 2D2 TCR transgenic mice (expressing a T cell receptor specific for the Myelin Oligodendrocyte Glycoprotein (MOG)_35–55_ peptide presented by I-A^b^) were maintained on a C57BL/6 background and bred in our facility (van Niel et al. 2002; Bettelli et al. 2003). All mice were maintained under specific pathogen-free facility (SPF) conditions at the animal facility of Kanazawa university.

### Plasmids construct

All constructs encoding AP-EVs components were cloned into either the pCAGGS plasmid backbone (a gift from Dr. Jun-ichi Miyazaki, Osaka University, Japan) or the pLJM1 plasmid backbone (Addgene, Plasmid #19319) via the multicloning site (MCS). For EV surface anchoring, mouse MHC class II *β* chains and Latency-Associated Peptide (LAP)–TGF-*β* were fused to the *N*-terminus of mouse CD81 or mouse MFG-E8 (D89E mutant). Mutations in the LAP domain of TGF-*β* (C33S, C223S, C225S) were introduced based on previous reports (Brunner et al. 1989; Zhang et al., 2010). The extracellular domain of mouse IL−2 was inserted into either the second extracellular loop of CD81 or the C-terminus of MFG-E8. The MHC class II *α* chain was cloned separately into the MCS to allow co-expression. For antigen loading, sequences encoding OVA_323-339_ (ISQAVHAAHAEINEAGR) or MOG_38-50_ (VGWYRSPFSRVVVHL) were genetically fused to the MHC class II *β* chain using either a (GGGGS)₂ linker or a GGGGGGTSGGGSGGS linker, respectively. The MFG-E8–based fusion constructs cloned into the pLJM1 vector were designed to co-express fluorescent proteins using P2A sequences for cell sorting by flow cytometry. Specifically, the MHC class II *α* chain was co-expressed with red fluorescent protein (RFP), the MHC class II *β* chain–MFG-E8 fusion with blue fluorescent protein (BFP), and the TGF-β–MFG-E8–IL−2 fusion protein with Venus.

### Generation of stable HEK293T cells expressing pMHCII, TGF-*β* and IL−2 fusion proteins

To generate HEK293T cells stably expressing OVA or MOG peptide–MHC class II complexes together with IL−2 and TGF-*β*, we employed a lentiviral transduction system. 1.0 × 10⁶ cells/well of Lenti-X 293 T cells were seeded into 6-well plates one day before transfection. Lentiviral particles were produced by co-transfecting the cells with 1 μg each of pRSV-Rev, pMD2.G, pMDLg/pRRE, and the transfer vector pLjM1 encoding the fusion constructs using PEI. 8 h after transfection, the medium was replaced with 1.5 mL of fresh culture medium. Viral supernatants were harvested at 24 and 48 h post-transfection, passed through a 0.45 μm syringe filter, and used immediately or stored at 4 °C. For transduction, HEK293T cells seeded the previous day were infected with the filtered viral supernatants mixed with DOTAP (Roche, Basel, Switzerland) at a 1:100 dilution. The viral mixture was added directly to the cells and centrifuged at 2,500 rpm for 2 h at 32 °C (accel 1, decel 3) to enhance infection efficiency. Following transduction, HEK293T cells expressing high levels of MHCII, IL−2, and TGF-*β* were enriched using a BD FACSMelody™ cell sorter (BD Biosciences, San Jose, CA, USA), based on fluorescent protein expression or surface marker levels.

### Western blot analysis

For cell lysate samples, HEK293T cells were lysed using RIPA buffer (50 mmol/L Tris-HCl, pH 8.0, 150 mM NaCl, 1% NP−40, 0.5% sodium deoxycholate, 0.1% SDS, and 2 mM EDTA) supplemented with protease inhibitor (Nacalai Tesque, Kyoto, Japan). For Western blotting, either 10 µg of total cell lysate protein or 1.0 × 10^8^ particles of EVs were used.

Primary antibodies used were: anti-human *β*-actin (AC−15, 1:1000, Sigma-Aldrich**,** St. Louis, MO, USA), anti-human CD81 (5A6, 1:1000, BioLegend, San Diego, CA, USA), anti-human CD9 (HI9a, 1:1000, BioLegend, San Diego, CA, USA). Secondary antibodies used were: HRP Goat anti-mouse IgG (Poly4053, 1:5000, BioLegend, San Diego, CA, USA) and HRP Goat anti-rat IgG (Poly4054, 1:5000, BioLegend, San Diego, CA, USA). All antibodies were diluted using Can Get Signal® Immunoreaction Enhancer Solution (TOYOBO, Osaka, Japan). Proteins were detected using SuperSignal™ West Dura Extended Duration Substrate (Thermo Fisher Scientific, Waltham, MA, USA) following the manufacturer’s protocol. Blots were imaged with the Fusion imaging system (Vilber, TechnoSaurus, Bern, Switzerland).

### Nanoparticle tracking analysis

The number and characteristics of AP-EVs were determined using a NanoSight LM10 (Malvern Panalytical, Malvern, UK). Briefly, 600 μL of diluted AP-EV solution was loaded onto the sample chamber. The particle movement was recorded for 30 seconds at a camera level setting of 15. Three different fields were captured for each sample to ensure reproducibility. Data were analyzed using NanoSight NTA software version 3.1 (Malvern Panalytical, Malvern, UK) with a detection threshold set to 3.

### Confocal microscopy

HEK293T cells were co-transfected with either CD81–RFP and CD81–GFP, or GFP–MFG-E8 and RFP–MFG-E8 constructs. EVs were then isolated from the culture supernatant by ultracentrifugation, as described in the section “Preparation and purification of AP-EVs.” The EV-containing supernatant was filtered through a 0.22-μm syringe filter and diluted to a final concentration of 2.0 × 10⁸ particles in 30 μL of PBS. A 30 μL aliquot was applied to a glass slide and covered with a coverslip. Fluorescent imaging was performed using a 100 × oil immersion objective lens on a Nikon A1R confocal microscope. Colocalization of GFP and RFP signals was analyzed and quantified using Imaris software (Oxford Instruments, Oxfordshire, UK).

### Single EV analysis

4.0 × 10^9^ particles of EVs were isolated by ultracentrifugation and subsequently labeled with fluorescently conjugated antibodies at a dilution of 1:10 in 40 µL of immobilization buffer provided in the MagCapture™ Exosome Isolation Kit PS Ver.2 (FUJIFILM Wako, Osaka, Japan). Labeling was performed at room temperature for 2 h. The labeled EV suspension was then diluted with 960 µL of immobilization buffer containing a binding enhancer and incubated with Tim4-conjugated magnetic beads for selective capture. Following bead binding, EVs were released by eluting twice with 40 µL of elution buffer for 10 min each. The purified, antibody-labeled EVs were analyzed using a Flow NanoAnalyzer (NanoFCM, Xiamen, China), equipped with 488 nm and 638 nm lasers and band-pass filters of 488/10, 525/40, and 670/40. All procedures were conducted in accordance with the manufacturer's instructions. Flow cytometric data were analyzed using FlowJo software (version 10.4.1, Ashland, OR, USA).

### Antibodies

Antibody staining was performed according to standard protocols. Monoclonal antibodies used for surface staining included the following: anti-mCD4 (GK1.5), mCD25 (PC61), mPD-L1 (10F.9G2), mCTLA−4 (UC10-4B9), mLAG−3 (C9B7W), mTim−3 (B8.2C12), mTIGIT (1G9), mTCR-Vα2 (B20.1), mTCR-Vβ5 (MR9−4), mTCR-Vα3.2 (RR3−16), mTCR-Vβ11 (KT11), mCD45.1 (A20), mCD45.2 (104), mMHCII (M5/114.15.2), mCD80 (16-10A1), mIL−2 (JES6-5H4), mTGF-*β* (TW7-16B4), mCD8 (53−6.7), mCD11b (M1/70), mCD11c (N418), mCD19 (6D5), mCD69 (H1.2F3), mCD86 (A17199A), mNK1.1 (S17016D), mLy6G (1A8) (all from BioLegend, San Diego, CA, USA).

Intracellular staining was performed using anti-mFoxp3 (MF−14) antibody, in combination with the True-Nuclear™ Transcription Factor Buffer Set (BioLegend, San Diego, CA, USA).

### Flow cytometric analysis of EVs

The surface expression of MHCII, CD80, TGF-*β*, and IL−2 on EVs was evaluated using the PS Capture™ Exosome Flow Cytometry Kit (FUJIFILM Wako, Osaka, Japan), following the manufacturer’s instructions. Briefly, supernatants collected after centrifugation at 10,000 × g for 30 min were further purified and incubated with EV-capture beads for 1 h at room temperature. The bead-bound EVs were then stained with fluorescently labeled antibodies (all diluted 1:200), vortexed every 20 min during incubation, and subsequently washed. Flow cytometric analysis was performed using a CytoFLEX flow cytometer (Beckman Colter, Brea, CA, USA), and data were analyzed using FlowJo software version 10.4.1.

### AFM analysis

EVs were isolated using the MagCapture™ Exosome Isolation Kit PS Ver.2 according to the manufacturer’s instructions. For AFM analysis, isolated EVs were immobilized on mica substrates that had been pre-treated with 0.01% APTES in Milli-Q water for 3 min. Following the incubation, the EV samples were applied onto the APTES-coated mica surfaces. High-speed atomic force microscopy (HS-AFM) was conducted in tapping mode using BL-AC10DS-A2 cantilevers. The spring constant of the cantilevers was determined using the thermal tuning method. AFM image rendering and nanomechanical data processing were performed using UMEX Viewer software (Kanazawa University, Ishikawa, Japan).

### *In vitro* T cell differentiation assay

Antigen-specific CD4⁺ T cells were isolated from OT-II, 2D2 transgenic mice, or wild-type (WT) mice and labeled with 1 μM Cell Trace™ Violet (CTV; Thermo Fisher Scientific, Waltham, MA, USA) at 37 °C for 3 min. Labeled cells were washed three times with complete culture medium to remove excess dye. A total of 2.0 × 10⁵ CTV-labeled T cells were then co-cultured with either AP-EVs or control EVs for 4 to 7 days. Unless otherwise specified, EVs were added at a working concentration of 2.0 × 10⁹ particles/mL. For the evaluation of Treg suppressive molecules, cultures were supplemented with 5 µg/mL anti-mouse CD3ε (BioLegend, San Diego, CA, USA), 10 ng/mL of recombinant mouse IL−2 (BioLegend, San Diego, CA, USA), and 10 ng/mL of TGF-*β* (BioLegend, San Diego, CA, USA). T cell proliferation and differentiation were assessed by flow cytometry.

### *In vitro* suppression assay

OVA-specific CD4⁺ T cells (CD45.1/2) were isolated from OT-II transgenic mice and co-cultured with 2.0 × 10⁹ particles/mL of AP-EVs for 4 days. OVA-specific Tregs induced by CD81-type AP-EVs, were co-cultured with responder OT-II T cells (CD45.2) at responder-to-Treg ratios of 1:1, 2:1, and 4:1. Each well contained 3.0 × 10⁵ responder T cells and 6.0 × 10⁴ stimulator cells (CD45.1). The stimulator cells were splenocytes isolated from CD45.1 congenic mice and pulsed with 3 μM OVA_323-339_ peptide. Prior to co-culture, only responder OT-II T cells, but not Tregs, were labeled with 1 μM CTV, and their proliferation was assessed by flow cytometry after 72 h.

### Adoptive T cell transfer

CD45.2⁺ T cells from OT-II or 2D2 transgenic mice were mixed at a 1:1 ratio with CD45.1⁺ T cells isolated from wild-type C57BL/6 mice. The mixed population was labeled with CTV, and a total of 2.0 × 10⁶ T cells were intravenously injected into CD45.1/2 congenic recipient mice. For EVs administration, two independent dosing regimens were employed. In the first regimen, mice received 5.0 × 10¹⁰ particles of either AP-EVs or control EVs on days 1 and 4 post-transfer via intravenous, intraperitoneal, or dorsal subcutaneous injection. In a separate regimen, mice were treated with the same dose of EVs once daily for six consecutive days following T cell transfer, administered through the intravenous injection. On day 7, spleens were harvested, and single-cell suspensions were prepared and analyzed by flow cytometry. 

### *In vivo* generation of Tregs by AP-EVs and mTOR inhibition

CD45.2⁺ T cells from OT-II transgenic mice were labeled with CTV, and a total of 2.0 ×  10⁶ T cells were intravenously injected into CD45.1/2 congenic recipient mice. On days 1 and 4 post-transfer, mice received 5.0 × 10¹⁰ particles of either AP-EVs or control EVs. In experiments involving mTOR inhibition, recipient mice were additionally administered rapamycin (2 mg/kg; Funakoshi, Tokyo, Japan) intraperitoneally once daily for four consecutive days, beginning one day after the initial EV treatment. On day 6 or 13, spleens were harvested, and single-cell suspensions were prepared for analysis. Treg differentiation and proliferation were evaluated by flow cytometry.

### Safety evaluation of EVs

 For short-term monitoring, mice were administered 5.0 × 10¹⁰ particles of AP-EVs or control EVs. At 24 h post-administration, spleens were harvested, and total splenocyte counts as well as activation markers of antigen-presenting cells and lymphocytes were analyzed by flow cytometry.

 For long-term monitoring, mice received 5.0 × 10¹⁰ particles of AP-EVs or control EVs every other day for a total of three injections. Body weight was recorded every other day or every two days throughout the observation period. Two weeks after the final injection, mice were anesthetized with a triple anesthetic cocktail. Mice were perfused, and the liver was fixed in 4% formalin at 4 °C overnight. After paraffin embedding, sections were cut at 3 μm thickness and stained with H&E. Paraffin embedding, sectioning, and H&E staining were conducted by the Technical Support Section of Kanazawa University (Kanazawa, Japan). All histological images were acquired using the BZ-X series all-in-one fluorescence microscope (KEYENCE, Osaka, Japan).

### Statistical analysis

One-way analysis of variance (ANOVA) was used to compare differences among multiple groups. For comparisons between two groups, unpaired two-tailed Student’s t-tests were performed. All statistical analyzes were performed with GraphPad Prism version 8.0 (GraphPad Software, San Diego, CA, USA). *P ≤ 0.05, **P ≤ 0.01, ***P ≤ 0.001, ****P ≤ 0.0001.

## Results

### Molecular characterization of AP-EVs

**Figure 1.**
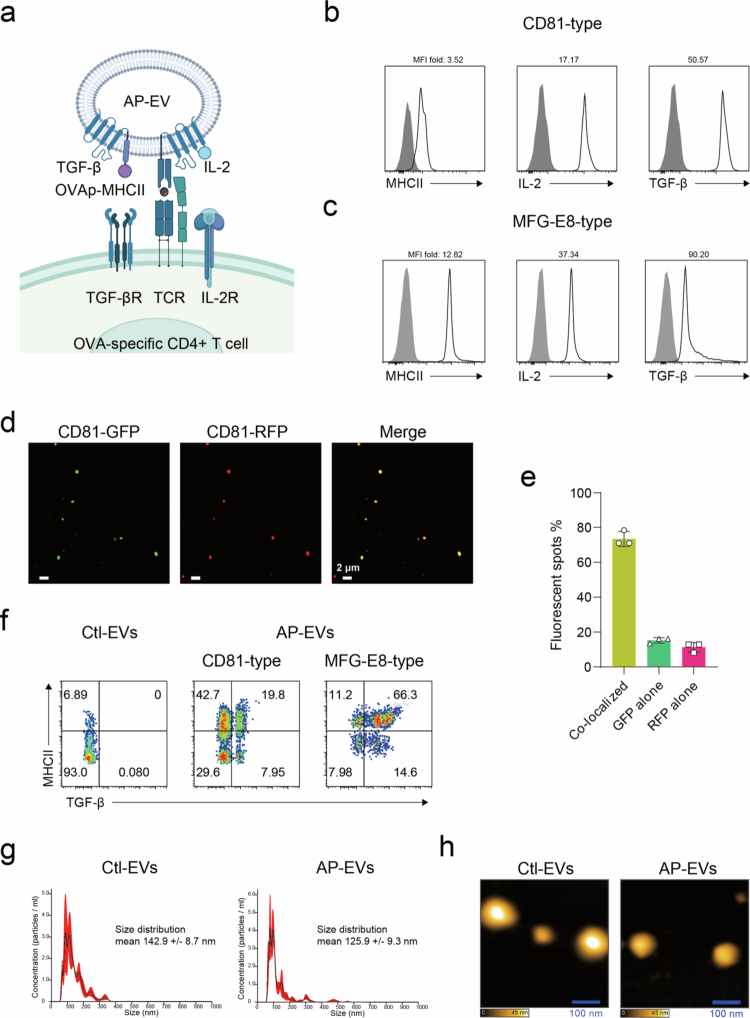
Molecular and biophysical characterization of AP-EVs. (a) Schematic representation of engineered antigen-presenting EVs (AP-EVs) displaying OVA peptide–MHC class II (OVAp-MHCII) complexes, IL−2, and TGF-*β*, which interact with the TCR, IL-2R, and TGF-βR, respectively, on antigen-specific CD4⁺ T cells. (b) CD81-type AP-EVs and control EVs were captured using Tim4-conjugated beads. Surface expression of MHCII, IL−2, and TGF-*β* was assessed by flow cytometry. Gray-filled histograms indicate control EVs; open black histograms indicate AP-EVs. The fold change in the mean fluorescence intensities (MFI) of MHCII, IL−2, and TGF-*β* relative to control EVs is also shown. (c) MFG-E8-type AP-EVs and control EVs were analyzed using the same method as in (b). (d, e) HEK293T cells were co-transfected with plasmids encoding CD81-RFP and CD81-GFP fusion proteins. EVs were purified by ultracentrifugation and analyzed by confocal microscopy. Scale bar indicates 2 μm. The number of dual-positive fluorescent dots (GFP⁺RFP⁺) was quantified. (f) AP-EVs were labeled with fluorescence-conjugated anti-MHCII and anti–TGF-β antibodies. Single EVs were analyzed using NanoFCM. (g) Size distribution of AP-EVs particles was assessed by nanoparticle tracking analysis (NTA) using the NanoSight LM10 system. Three independent fields were recorded and analyzed. (h) Morphology of AP-EVs and control EVs was visualized by atomic force microscopy (AFM).

IL−2, TGF-*β*, and antigenic stimulation are the most established and synergistic factors for Treg induction in both experimental and clinical contexts (Gu et al., 2022). To develop extracellular vesicles capable of inducing antigen-specific regulatory T cells (Tregs), we engineered a new class of antigen-presenting EVs, termed AP-EVs, to co-display pMHCII, IL−2, and TGF-*β* on their surface. To activate TGF-β1 in a membrane-bound context, we generated a constitutively active form of TGF-β1. The pro-domain of the TGF-β1 precursor contains three conserved cysteine residues (C33, C223, C225) that mediate interchain disulfide bonding and prevent the release of mature cytokine (Brunner et al. 1989; Zhang et al., 2010). We mutated all three cysteines to serine (C33S, C223S, C225S), which enabled constitutive activity of TGF-β1 upon EV expression. These immunomodulatory components were fused to CD81, a tetraspanin commonly used as an EV membrane anchor (Figure 1a, Figure. S1a-c) (Stickney et al., 2016). As an alternative strategy, we also designed constructs in which the same set of molecules was fused to milk fat globule-EGF factor 8 (MFG-E8), a glycoprotein that facilitates membrane localization (Delcayre 2005; Dooley 2021). To reduce phagocytic clearance, a D89E point mutation was introduced into MFG-E8, which has been reported to inhibit phosphatidylserine recognition by phagocytes (Figure. S1d-f) (Asano 2004). EVs were isolated from the supernatants of HEK293T cells stably expressing either CD81-based or MFG-E8–based fusion proteins. Flow cytometry analysis confirmed that the engineered EVs successfully displayed Ovalbumin (OVA) pMHCII, IL−2, and TGF-*β* on their surface (Figure 1b, c). Western blot analysis confirmed the absence of intracellular contaminants such as human *β*-actin, verifying the purity of the EV preparation (Figure. S2a).

To validate the co-display of multiple proteins on the same EV and to ensure that fusion constructs were incorporated as intended, HEK293T cells were co-transfected with plasmids encoding either CD81 fused with green fluorescent protein (GFP) and red fluorescent protein (RFP), or MFG-E8 fused with GFP and RFP. EVs were subsequently isolated from culture supernatants and analyzed by confocal microscopy to confirm the co-localization of fluorescent signals on individual vesicles. Approximately 80% of CD81-type EVs and 90% of MFG-E8-type EVs co-expressed both GFP and RFP (Figure 1d, e; Figure. S2b, c), indicating that the majority of EVs display multiple proteins simultaneously, regardless of the anchoring strategy. These findings are consistent with previous reports demonstrating the feasibility of multi-protein loading onto individual EVs (Corso et al., 2019; Lyu et al., 2025). While the confocal analysis confirmed the co-localization of fluorescent protein tags as a proxy for co-expression, it remained unclear whether functional immunoregulatory molecules such as IL−2 and TGF-*β* were also co-present on individual EVs. To directly evaluate this, we employed an advanced flow cytometry platform capable of single EV analysis, NanoFCM. Single-EV analysis demonstrated the presence of the engineered fusion proteins MHCII–CD81–IL−2 and TGF-β–CD81 on individual vesicles. Co-expression of MHCII and TGF-*β* was observed in approximately 20% of CD81-type EVs and over 60% of MFG-E8-type EVs (Figure 1f).

Nanoparticle tracking analysis revealed that the average size of CD81-type AP-EVs was approximately 150 nm, comparable to unmodified control EVs (Figure 1g). Atomic force microscopy (AFM) showed that CD81-type AP-EVs exhibited size and shape comparable to those of control EVs (Figure 1h; Figure. S2d).

### AP-EVs induce antigen-specific Tregs *in vitro*

**Figure 2.**
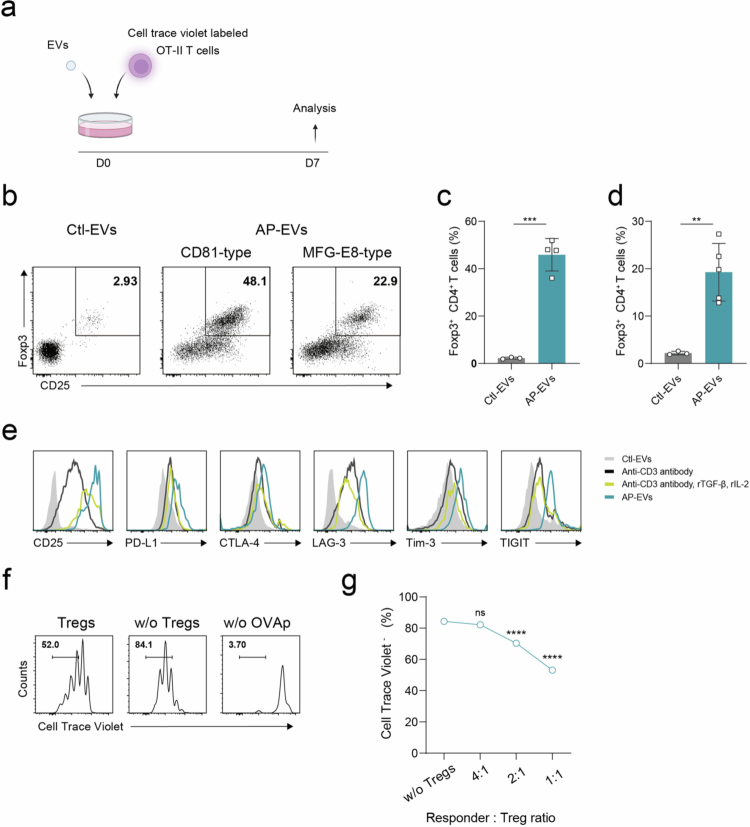
*In vitro* induction of antigen-specific regulatory T cells by AP-EVs. (a) OVA-specific CD4⁺ T cells from OT-II transgenic mice were labeled with 1 μM CTV. A total of 2.0 × 10⁵ CTV-labeled cells were co-cultured with either AP-EVs or control EVs at a concentration of 2.0 × 10⁹ particles/mL for 7 days. (b–d) Treg differentiation was assessed by CD25 and Foxp3 expression using flow cytometry. (e) OT-II T cells were cultured for four days under three different conditions: with AP-EVs, with anti-CD3 antibody alone, or with anti-CD3 antibody in combination with recombinant IL−2 and recombinant TGF-*β* at a concentration of 10 ng/mL respectively. Expression of CD25, PD-L1, CTLA−4, LAG−3, TIM−3, and TIGIT on Foxp3⁺ cells were evaluated by flow cytometry. (f) OVA-specific Tregs (CD45.1/2), induced by CD81-type AP-EVs, were co-cultured with responder OT-II T cells (CD45.2) and OVA-pulsed stimulator splenocytes (CD45.1) for 72 h. CTV was used to label responder OT-II T cells, not Tregs. Responder T cell proliferation was evaluated by CTV dilution using flow cytometry. (g) Suppression assay using responder:Treg ratios of 1:1, 2:1, and 4:1. Proliferation of responder OT-II T cells was assessed after 72 h of co-culture. Statistical comparisons were performed between each Treg-treated condition and the no-Treg control group. All statistical analyzes were performed with GraphPad Prism version 8.0. *P ≤ 0.05, **P ≤ 0.01, ***P ≤ 0.001, ****P ≤ 0.0001.

To explore the potential of EVs to induce antigen-specific Tregs, we evaluated whether OVA peptide-presenting AP-EVs could induce antigen-specific Tregs *in vitro*. OVA-specific CD4⁺ T cells (OT-II T cells) were isolated from the lymph nodes of OT-II TCR transgenic mice and labeled with Cell Trace Violet (CTV). The labeled OT-II T cells were then cultured with either control EVs or AP-EVs at a concentration of 2.0 × 10⁹ particles/mL for various durations. After 4 or 7 days of culture, we assessed Treg differentiation (Figure 2a). While Treg induction was negligible with control EVs, co-culture with AP-EVs led to robust differentiation of OT-II T cells into Tregs by both CD81-type and MFG-E8-type AP-EVs (Figure 2b–d). Furthermore, the frequency of Treg conversion increased by day 7 compared to day 4 (Figure. S3a, b), and the majority of Tregs were CTV⁻, consistent with multiple rounds of cell division (Figure. S3c). The induction of antigen-specific Treg cells by AP-EVs increased in a dose-dependent manner (Figure. S3d), indicating that AP-EVs efficiently promote the differentiation of naïve OT-II T cells.

We next examined the expression of immunoregulatory molecules typically associated with Tregs. Antigen-specific Tregs induced by AP-EVs expressed high levels of CD25, PD-L1, CTLA−4, LAG−3, Tim−3, and TIGIT, all of which are hallmarks of Treg-mediated immune regulation (Figure 2e). To assess their suppressive capacity, we performed co-culture assays using OVA peptide-loaded splenocytes as stimulators. OT-II Tregs were co-cultured with responder OT-II T cells at varying ratios. The induced OT-II Tregs effectively suppressed responder T cell proliferation in a ratio-dependent manner (Figure 2f, g). Notably, the extent of suppression increased with the proportion of induced Tregs, demonstrating a typical dose-dependent suppression pattern. In line with their expression of multiple checkpoint and inhibitory molecules, these results suggest that AP-EV-induced Tregs suppress immune responses through both contact-dependent and cytokine-mediated mechanisms, consistent with canonical Treg biology. Collectively, these findings indicate that AP-EVs can efficiently induce functional, antigen-specific Tregs *in vitro*.

### MOG-specific Treg induction by AP-EVs in 2D2 TCR transgenic mice

**Figure 3.**
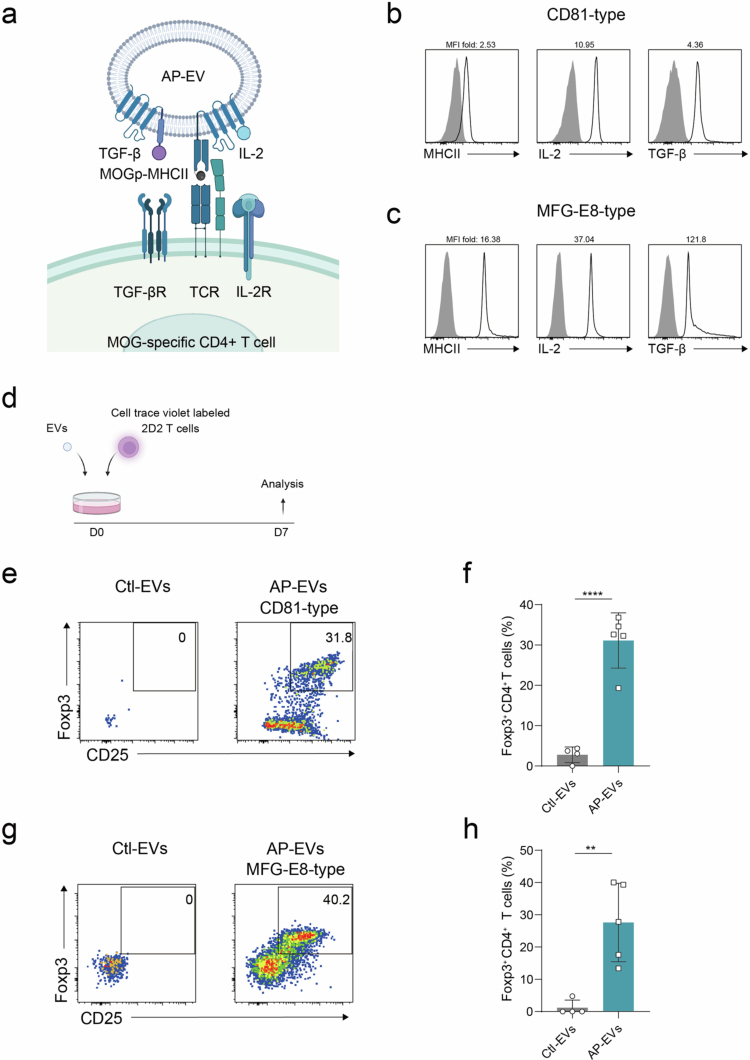
**Antigen-specific Treg induction by MOG-loaded AP-EVs.** (a) Schematic representation of AP-EVs displaying MOG peptide–MHC class II (MOGp-MHCII) complexes, IL−2, and TGF-*β*. (b) CD81-type AP-EVs and control EVs were captured using Tim4-conjugated beads. Surface expression of MHCII, IL−2, and TGF-*β* was assessed by flow cytometry. Gray-filled histograms indicate control EVs; open black histograms indicate AP-EVs. The fold change in the MFI of MHCII, IL−2, and TGF-*β* relative to control EVs is also shown. (c) MFG-E8-type AP-EVs and control EVs were analyzed using the same method as in (b). (d) MOG-specific CD4⁺ T cells (2D2) isolated from 2D2 transgenic mice were labeled with 1 μM CTV. 2.0 × 10⁵ CTV-labeled 2D2 T cells were co-cultured with either AP-EVs or control EVs for 7 days. (e, f) Treg induction (CD25⁺Foxp3⁺) of 2D2 T cells co-cultured with CD81-type AP-EVs at a concentration of 2.0 × 10⁹ particles/mL were evaluated by flow cytometry. (g, h) Treg induction of 2D2 T cells co-cultured with MFG-E8-type AP-EVs were similarly analyzed. All statistical analyzes were performed with GraphPad Prism version 8.0. *P ≤ 0.05, **P ≤ 0.01, ***P ≤ 0.001, ****P ≤ 0.0001.

To extend the applicability of AP-EVs to autoimmune disease models, we constructed AP-EVs particles expressing MHC II loaded with a myelin oligodendrocyte glycoprotein (MOG) peptide (Figure 3a). Although the known epitope recognized by 2D2 T cells is MOG_35-55,_ we observed reduced surface expression of the pMHCII when this peptide was used. To address this, we optimized the linker sequence and selected the MOG_38-50_ peptide for efficient EV loading (Figure. S4a-f) (Rosenthal et al., 2012). This strategy enabled the successful generation of CD81-type and MFG-E8-type AP-EVs particles that highly expressed MOG_38-50_-MHCII, IL−2, and TGF-*β* (Figure 3b, c).

To assess whether these MOG peptide-expressing AP-EVs could induce antigen-specific Tregs *in vitro*, we isolated T cells from the lymph nodes of 2D2 transgenic mice and labeled them with CTV. The labeled cells were cultured with either control EVs or AP-EVs at a concentration of 2.0 × 10^9^ particles/mL. After 4 or 7 days of culture, we assessed the differentiation of 2D2 T cells into Tregs (Figure 3d). Minimal Treg induction was observed in cultures with control EVs, whereas co-culture with AP-EVs resulted in a robust increase in Foxp3⁺ Tregs induced by both CD81-type and MFG-E8-type AP-EVs (Figure 3e–h). Furthermore, Treg induction was greater at day 7 compared to day 4, consistent with progressive expansion of the Treg population (Figure. S5a, b). These results indicate that CD81-type and MFG-E8-type AP-EVs effectively induced Foxp3⁺ regulatory T cells from autoreactive CD4⁺ T cells derived from 2D2 transgenic mice.

### Enhancement of *In Vivo* Treg Induction by AP-EVs through mTOR Inhibition

We next investigated whether AP-EVs could induce antigen-specific Tregs *in vivo*. Lymphocytes were isolated from CD45.2 OT-II mice and CD45.1 wild-type mice, labeled with CTV, and adoptively transferred into CD45.1/2 recipient mice. On days 1 and 4 post-transfer, the mice were administered 5.0 × 10^10^ particles of either control EVs or AP-EVs. On day 7, spleens were harvested, and the proliferation and Treg differentiation of donor cells were analyzed (Figure. S6a). In mice treated with control EVs, neither wild-type nor OT-II T cells showed signs of proliferation. In contrast, in mice treated with AP-EVs, proliferation was observed exclusively in OT-II T cells (Figure. S6b–d). Similar results were obtained using 2D2 T cells (Figure. S6e). These findings suggest that AP-EVs are selectively delivered to antigen-specific T cells *in vivo* and can activate them in an antigen-dependent manner. However, despite this selective activation, neither Foxp3 nor CD25 expression was detected in OT-II T cells, suggesting that AP-EVs alone is insufficient to induce Treg differentiation *in vivo* (Figure. S6f).

To enhance Treg induction, we tested several modifications, including increasing the frequency of AP-EV administration and altering the route of delivery. However, none of these approaches led to detectable Foxp3 induction (Figure. S6g, h).

**Figure 4.**
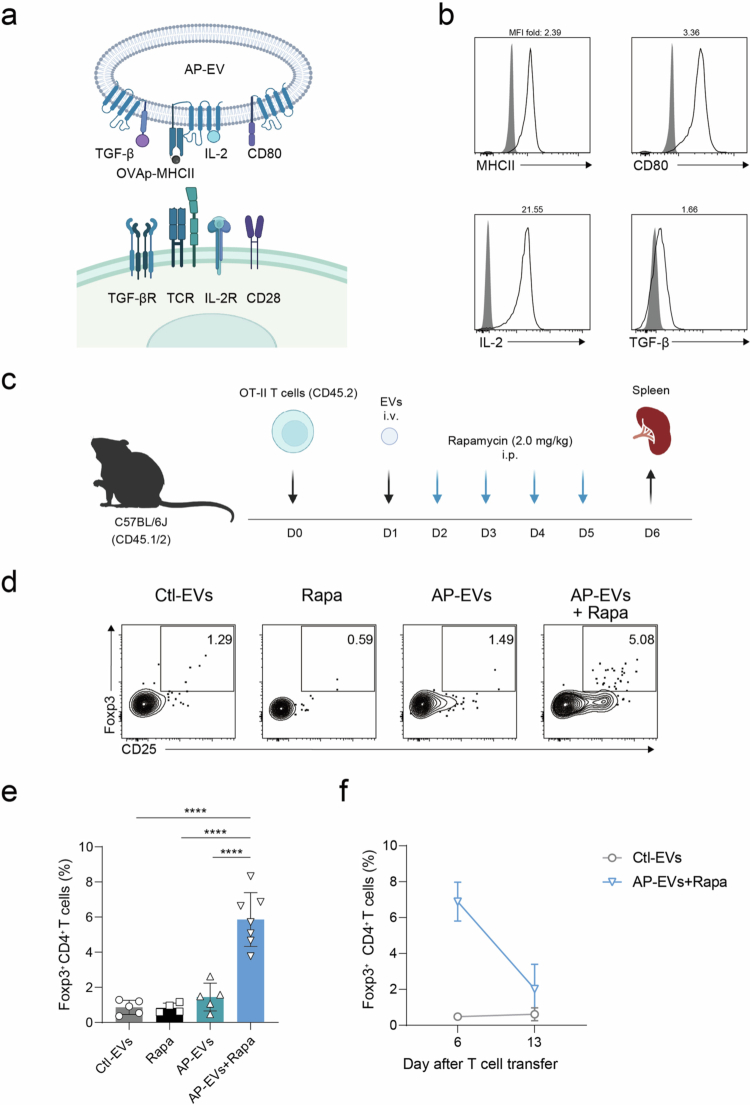
***In vivo*** **induction of Tregs by AP-EVs and mTOR inhibition.** (a) Schematic representation of engineered antigen-presenting EVs (AP-EVs) displaying OVAp-MHCII, CD80, IL−2, and TGF-*β*, which interact with the TCR, CD28, IL-2R, and TGF-βR, respectively, on antigen-specific CD4⁺ T cells. (b) Surface expression of MHCII, CD80, TGF-*β*, and IL−2 on EVs was assessed by flow cytometry. The fold change in the MFI of MHCII, IL−2, and TGF-*β* relative to control EVs is also shown. (c) Schematic of the adoptive transfer and treatment protocol. (d, e) On day 6, spleens were harvested, and Treg differentiation was analyzed by flow cytometry (*n* = 4−7). (f) Induced Tregs in the spleen were further analyzed on day 13 after T cell transfer (*n* = 3−4). All statistical analyzes were performed with GraphPad Prism version 8.0. *P ≤ 0.05, **P ≤ 0.01, ***P ≤ 0.001, ****P ≤ 0.0001.

Given the importance of co-stimulation in Treg differentiation, we next engineered a modified version of AP-EVs expressing the co-stimulatory molecule CD80 in addition to the existing components (Figure 4a, b). Moreover, the Akt and mTOR pathways are known negative regulators of Foxp3 expression (Merkenschlager and von Boehmer, 2010). Inhibitors of these pathways, such as LY294002 (a PI3K/Akt inhibitor) and rapamycin (an mTOR inhibitor), have been reported to enhance Foxp3 induction (Sauer et al., 2008; Wang et al., 2020). To evaluate their effect in combination with AP-EVs, we examined Foxp3 expression *in vivo* following treatment (Figure 4c). Although CD80-expressing AP-EVs successfully induced CD25 expression in OT-II T cells, they failed to upregulate Foxp3. In contrast, the combination of AP-EVs with the mTOR inhibitor rapamycin significantly enhanced Foxp3 expression in OT-II T cells (Figure 4d, e). These results demonstrate that AP-EVs alone can activate antigen-specific T cells *in vivo*. However, co-administration of an mTOR inhibitor is required to promote their differentiation into Tregs. Interestingly, the induced Tregs in the spleen exhibited a tendency to decline within approximately one week after their induction, suggesting that the stability and persistence of AP-EV–induced Tregs may be limited (Figure 4f). Thus, while AP-EVs represent a promising platform for the induction of antigen-specific T cell responses, additional strategies will be required to ensure the stable and durable generation of functional Tregs for therapeutic applications.

### *In vivo* immunogenicity and safety assessment of HEK293T-derived EVs

To evaluate the immunogenicity and potential toxicity of HEK293T cell–derived EVs, mice were administered 5.0 × 10 ¹ ⁰ particles of AP-EVs or control EVs. At 24 h post-administration, spleens were harvested, and total splenocyte counts as well as activation markers on antigen-presenting cells and lymphocytes were analyzed. No significant increases in splenocyte numbers or in the abundance of immune cell populations were observed compared with untreated mice (Figure. S7a). Furthermore, early activation markers on T cells and NK cells (CD69), as well as co-stimulatory molecule expression on antigen-presenting cells, were not elevated following EV administration (Figure. S7b).

 To investigate potential long-term effects, mice received 5.0 × 10¹⁰ particles of AP-EVs or control EVs for 3 times, and body weight was monitored over 20 days. EV-treated mice showed no significant weight loss and displayed a similar weight gain trajectory as untreated mice (Figure. S7c). Histopathological analysis of the liver revealed no evidence of inflammatory cell infiltration or tissue damage, and no significant differences were detected compared with the untreated group (Figure. S7d).

Collectively, these results indicate that HEK293T-derived EVs, including AP-EVs, are well tolerated *in vivo* and do not induce measurable immune activation or organ toxicity under the conditions tested.

## Discussion

In this study, we developed engineered extracellular vesicles (AP-EVs) presenting pMHCII together with IL−2 and TGF-*β* to induce antigen-specific immune modulation. *In vitro*, AP-EVs alone were sufficient to induce Foxp3⁺ Tregs, demonstrating their intrinsic tolerogenic potential. *In vivo*, AP-EVs activated and expanded antigen-specific T cells, as indicated by increased CD25 expression, but Foxp3 induction required co-administration of rapamycin, highlighting the importance of mTOR inhibition under physiological conditions. These results suggest that while AP-EVs provide the necessary signals for Treg differentiation, the *in vivo* environment contains competing activation cues that limit their effect. Future strategies may include incorporating inhibitory molecules such as PD-L1 into AP-EVs to mimic rapamycin’s effects via PD−1 signaling and enable Treg induction without systemic drug administration.

We compared two strategies for displaying immune regulatory molecules on EVs: fusion to CD81 or MFG-E8. MFG-E8–type EVs had higher surface expression, while CD81-type EVs were slightly more effective for inducing antigen-specific Tregs *in vitro*. NanoFCM analysis showed that about 20% of CD81-type and over 60% of MFG-E8-type EVs were double-positive for MHCII and TGF-*β* (Figure 1f), whereas confocal microscopy detected much higher co-expression. This discrepancy likely reflects technical limits of antibody-based detection rather than actual absence of fusion proteins. Both EV types promoted Treg induction, but the optimal platform may vary depending on antigen specificity, biodistribution, and disease context, and further *in vivo* evaluation will be required to identify the most suitable design for clinical translation.

Several strategies have been developed to induce antigen-specific immune tolerance, including mRNA-based vaccines (Krienke et al., 2021; Wardell & Levings, 2021), adoptive transfer of Tregs, and nanoparticle-based systems. mRNA vaccines offer flexibility and transient expression but face challenges such as off-target effects, immunogenicity, and short half-life (Pardi et al., 2018). Adoptive Treg therapies are effective but require *ex vivo* expansion and carry risks of polyclonal immunosuppression (Duffy et al., 2019; Mohammadi et al., 2021). pMHCII-coated nanoparticles have induced TR1-like cells in autoimmune models (Clemente-Casares et al. 2016). However, the synthetic nature of nanoparticles raises concerns regarding biocompatibility, unintended immune activation, and toxicity in clinical settings (Kyriakides et al., 2021; Sau et al., 2024).

To address these challenges, EV-based delivery provides a cell-free, biocompatible platform with high stability and targeting capacity (Cheng and Hill 2022). For example, engineered EVs presenting peptide–HLA class I complexes with co-stimulatory or co-inhibitory molecules can modulate CD8⁺ T cell responses in a type 1 diabetes model (Becker 2023). Previously, we showed that AP-EVs displaying pMHCII with cytokines such as IL−2, IL−12, or IL−4 can expand antigen-specific T cells and enhance anti-tumor immunity (Kimura et al., 2025; Lyu et al., 2025). In this study, we applied this approach to induce immune tolerance by converting pathogenic CD4⁺ T cells into Foxp3⁺ Tregs, thereby extending the therapeutic potential of AP-EVs from cancer to autoimmune and allergic diseases.

In summary, we present a novel strategy for antigen-specific Treg induction using engineered EVs in combination with mTOR inhibition. This approach offers a tunable, scalable, and cell-free platform for immune modulation. Future studies should focus on optimizing EV design, assessing the long-term stability and suppressive function of AP-EV-induced Tregs, and evaluating the safety of repeated administration to fully realize the clinical applicability of this technology.

## Supplementary Material

Supplementary Material Supplementary Material

## Data Availability

Upon reasonable request, data can be obtained from the corresponding author.
