# Supplementary material for: Induction of antigen-specific regulatory T cells by engineered extracellular vesicles
Source: Drug Deliv. 2025 Dec 18;32(1):2586305. doi: 10.1080/10717544.2025.2586305 (PMC12716476; doi:10.1080/10717544.2025.2586305)
Supplement: Supplementary Material [file IDRD_A_2586305_SM7018.pdf]

a I-Aα amino acid sequence

|← Signal peptide →|← I-Aα
MPRSRALILGVLALTTMLSLCGGEDDIEADHVGTYGISVYQSPGDIGQYTFEFDGDELFYVDLDKKETVWMLPEFGQLASFDPQGGL
QNIAVVKHNLGVLTKRSNSTPATNEAPQATVFPKSPVLLGQPNTLICFVDNIFPPVINITWLRNSKSVADGVYETSFFVNRDYSFHKLS
I-Aα →|
YLTFIPSDDDIYDCKVEHWGLEEPVLKHWEPEIPAPMSELTETVVCALGLSVGLVGIVVGTIFIIQGLRSGGTSRHPGPL

b OVA-peptide-I-Aβ-CD81-IL-2 amino acid sequence

|← Signal peptide →|← OVA-peptide →|← Linker →|← I-Aβ
MALQIPSLLLSAAVVVLMVLSSPGTEGISQAVHAAHAEINEAGRGGGGSGGGGSGGDSERHFVYQFMGECYFTNGTQRIRYVTRYI
YNREEYVRYDSDVGEHRAVTELGRPDAEYWNSQPEILERTRAELDTVCRHNYEGPETHTSLRRLEQPNVVISLSRTEALNHHNTLV
CSVTDFYPAKIKVRWFRNGQEETVGVSSTQLIRNGDWTFQVLVMLEMTPRRGEVYTCHVEHPSLKSPITVEWRAQSESAWSKMLS
I-Aβ →|← CD81
GIGGCVLGVIFLGLGLFIRHRSQKGPRGPPPAGLLQMGVEGCTKCIKYLLFVFNFVFWLAGGVILGVALWLRHDPQTTSLLYLELGNK
PAPNTFYVGIYILIAVGAVMMFVGFLGCYGAIQESQCLLGTFFTCLVILFACEVAAGIWGFVNKDQIAKDVKQFYDQALQQAVMDDDA
CD81 →|←Linker →|← IL-2
NNAKAVVKTFHETLNCCGSNALTTLTTTILRNSLCPSGGGGSAPTSSSTSSSTAEAQQQQQQQQQQQHLEQLLMDLQELLSRME
NYRNLKLPRMLTFKFYLPKQATELKDLQCLEDELGPLRHVLDLTQSKSFQLEDAENFISNIRVTVVKLKGSDNTFECQFDDESATVVD
IL-2 →|←Linker →|← CD81 →|
FLRRWIAFCQSIISTSPQGGGGSGGNILTPLLQQDCHQKIDELFSGKLYLIGIAAIVVAVIMIFEMILSMVLCCGIRNSSVY

c TGF-β-CD8-CD81 amino acid sequence

|← Signal peptide →|← LAP domain
MPPSGLRLLPLLLPLPWLLVLTPGRPAAGLSTSKTIDMELVKRKRIEAIRGQILSKLRLASPPSQGEVPPGPLPEAVLALYNSTRDRVA
GESADPEPEPEADYYAKEVTRVLMVDRNNAIYEKTKDISHSIYMFFNTSDIREAVPEPPLLSRAELRLQRLKSSVEQHVELYQKYSNN
SWRYLGNRLLTPTDTPEWLSFDVTGVVRQWLNQGDGIQGFRFSAHSSSDSKDNKLHVEINGISPKRRGDLGTIHDMNRPFLLLMAT
LAP domain →|← TGF-β
PLERAQHLHSSRHRRALDTNYCFSSTEKNCCVRQLYIDFRKDLGWKWIHEPKGYHANFCLGPCPYIWSLDTQYSKVLALYNQHNPG
TGF-β →|← CD8
ASASPCCVPQALEPLPIVYYVGRKPKVEQLSNMIVRSCKCSKPQAPELRIFPKKMDAELGQKVDLVCEVLGSVSQGCSWLFQNSSS
KLPQPTFVVYMASSHNKITWDEKLNSSKLFSAMRDTNNKYVLTLNKFSKENEGYYFCSVISNSVMYFSSVVPVLQKVNSTTTKPVLRT
CD8 →|← CD81
PSPVHPTGTSQPQRPEDCRPRGSVKGTGLDFACDIYIWAPLAGICVALLLSLIITLICYHRSRKRVCKCPRPLVRQEGKPRPSEKIVM
GVEGCTKCIKYLLFVFNFVFWLAGGVILGVALWLRHDPQTTSLLYLELGNKPAPNTFYVGIYILIAVGAVMMFVGFLGCYGAIQESQCL
LGTFFTCLVILFACEVAAGIWGFVNKDQIAKDVKQFYDQALQQAVMDDDANNAKAVVKTFHETLNCCGSNALTTLTTTILRNSLCPSG
CD81 →|
GNILTPLLQQDCHQKIDELFSGKLYLIGIAAIVVAVIMIFEMILSMVLCCGIRNSSVY

d I-Aα-leucine zipper amino acid sequence

|← Signal peptide →|← I-Aα
MPRSRALILGVLALTTMLSLCGGEDDIEADHVGTYGISVYQSPGDIGQYTFEFDGDELFYVDLDKKETVWMLPEFGQLASFDPQG
GLQNIAVVKHNLGVLTKRSNSTPATNEAPQATVFPKSPVLLGQPNTLICFVDNIFPPVINITWLRNSKSVADGVYETSFFVNRDYSF
I-Aα →|←Linker
HKLSYLTFIPSDDDIYDCKVEHWGLEEPVLKHWEPEIPAPMSELTETVVCALGLSVGLVGIVVGTIFIIQGLRSGGTSRHPGPLGGG
Linker →|← Leucine zipper →|
GSLEIRAAFLRQRNTALRTEVAELEQEVQRLENEVSQYETRYGPLGGGK

e OVA-peptide-I-Aβ-leucine zipper-MFG-E8 amino acid sequence

|← Signal peptide →|← OVA-peptide →|← Linker →|← I-Aβ
MALQIPSLLLSAAVVVLMVLSSPGTEGISQAVHAAHAEINEAGRGGGGSGGGGSGGDSERHFVYQFMGECYFTNGTQRIRYVTRYI
YNREEYVRYDSDVGEHRAVTELGRPDAEYWNSQPEILERTRAELDTVCRHNYEGPETHTSLRRLEQPNVVISLSRTEALNHHNTLV
I-Aβ →|←Linker
CSVTDFYPAKIKVRWFRNGQEETVGVSSTQLIRNGDWTFQVLVMLEMTPRRGEVYTCHVEHPSLKSPITVEWRAQSESAWSKGGG
Linker →|← Leucine zipper →|←Linker →|← MFG-E8
GSLEIEAAFLERENTALETRVAELRQRVQRLRNRVSQYRTRYGPLGGGKGGGGSASGDFCDSSLCLNGGTCLTGQDNDIYCLCPE
GFTGLVCNETERGPCSPNPCYNDAKCLVTLDTQRGEIFTEYICQCPVGYSGIHCETETNYYNLDGEYMFTTAVPNTAVPTPAPTPDL
SNNLASRCSTQLGMEGGAIADSQISASSVYMGFMGLQRWGPELARLYRTGIVNAWTASNYDSKPWIQVNLLRKMRVSGVMTQGAS
RAGRAEYLKTFKVAYSLDGRKFEFIQDESGGDKEFLGNLDNNSLKVNMFNPTLEAQYIKLYPVSCHRGCTLRFELLGCELHGCSEPL
GLKNNTIPDSQMSASSSYKTWNLRAFGWYPHLGRLDNQGKINAWTAQSNSAKEWLQVDLGTQRQVTGIITQGARDFGHIQYVASYK
MFG-E8 →|
VAHSDDGVQWTVYEEQGSSKVFQGNLDNNSHKKNIFEKPFMARYVRVLPVSWHNRITLRLELLGC

f TGF-β-MFG-E8-IL-2 amino acid sequence

|← Signal peptide →|← LAP domain
MPPSGLRLLPLLLPLPWLLVLTPGRPAAGLSTSKTIDMELVKRKRIEAIRGQILSKLRLASPPSQGEVPPGPLPEAVLALYNSTRDRVA
GESADPEPEPEADYYAKEVTRVLMVDRNNAIYEKTKDISHSIYMFFNTSDIREAVPEPPLLSRAELRLQRLKSSVEQHVELYQKYSNN
SWRYLGNRLLTPTDTPEWLSFDVTGVVRQWLNQGDGIQGFRFSAHSSSDSKDNKLHVEINGISPKRRGDLGTIHDMNRPFLLLMAT
LAP domain →|← TGF-β
PLERAQHLHSSRHRRALDTNYCFSSTEKNCCVRQLYIDFRKDLGWKWIHEPKGYHANFCLGPCPYIWSLDTQYSKVLALYNQHNPG
TGF-β →|←Linker →|← MFG-E8
ASASPCCVPQALEPLPIVYYVGRKPKVEQLSNMIVRSCKCSGGGGSASGDFCDSSLCLNGGTCLTGQDNDIYCLCPEGFTGLVCNE
TERGPCSPNPCYNDAKCLVTLDTQRGEIFTEYICQCPVGYSGIHCETETNYYNLDGEYMFTTAVPNTAVPTPAPTPDLSNNLASRCS
TQLGMEGGAIADSQISASSVYMGFMGLQRWGPELARLYRTGIVNAWTASNYDSKPWIQVNLLRKMRVSGVMTQGASRAGRAEYLK
TFKVAYSLDGRKFEFIQDESGGDKEFLGNLDNNSLKVNMFNPTLEAQYIKLYPVSCHRGCTLRFELLGCELHGCSEPLGLKNNTIPDS
QMSASSSYKTWNLRAFGWYPHLGRLDNQGKINAWTAQSNSAKEWLQVDLGTQRQVTGIITQGARDFGHIQYVASYKVAHSDDGVQ
MFG-E8 →|←Linker →|← IL-2
WTVYEEQGSSKVFQGNLDNNSHKKNIFEKPFMARYVRVLPVSWHNRITLRLELLGCGGGGSAPTSSSTSSSTAEAQQQQQQQQQ
QQHLEQLLMDLQELLSRMENYRNLKLPRMLTFKFYLPKQATELKDLQCLEDELGPLRHVLDLTQSKSFQLEDAENFISNIRVTVVKLKG
IL-2 →|
SDNTFECQFDDESATVVDFLRRWIAFCQSIISTSPQ

Supplementary Figure 1. Imai S, et al.

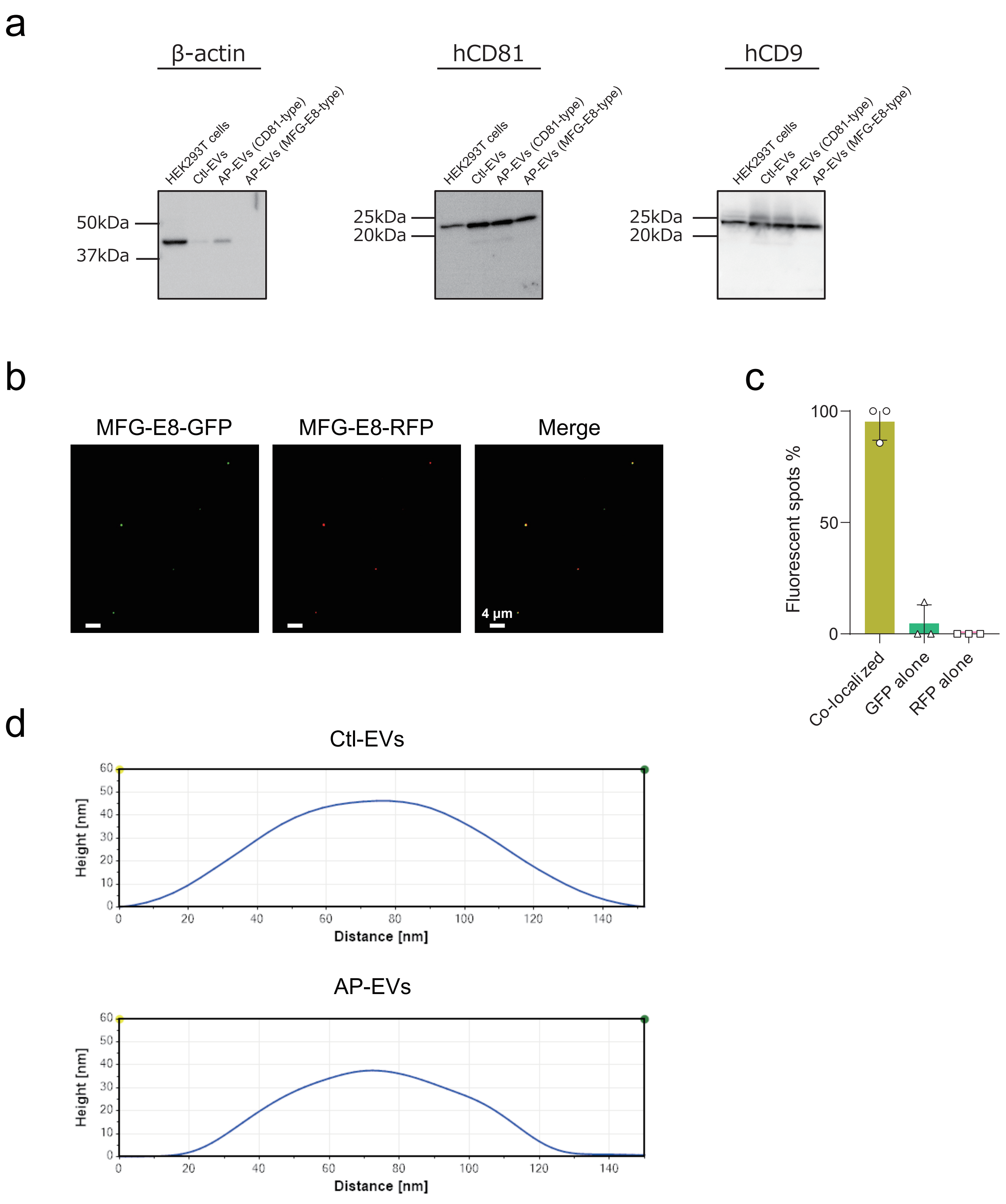

Supplementary Figure 2. Imai S, et al.

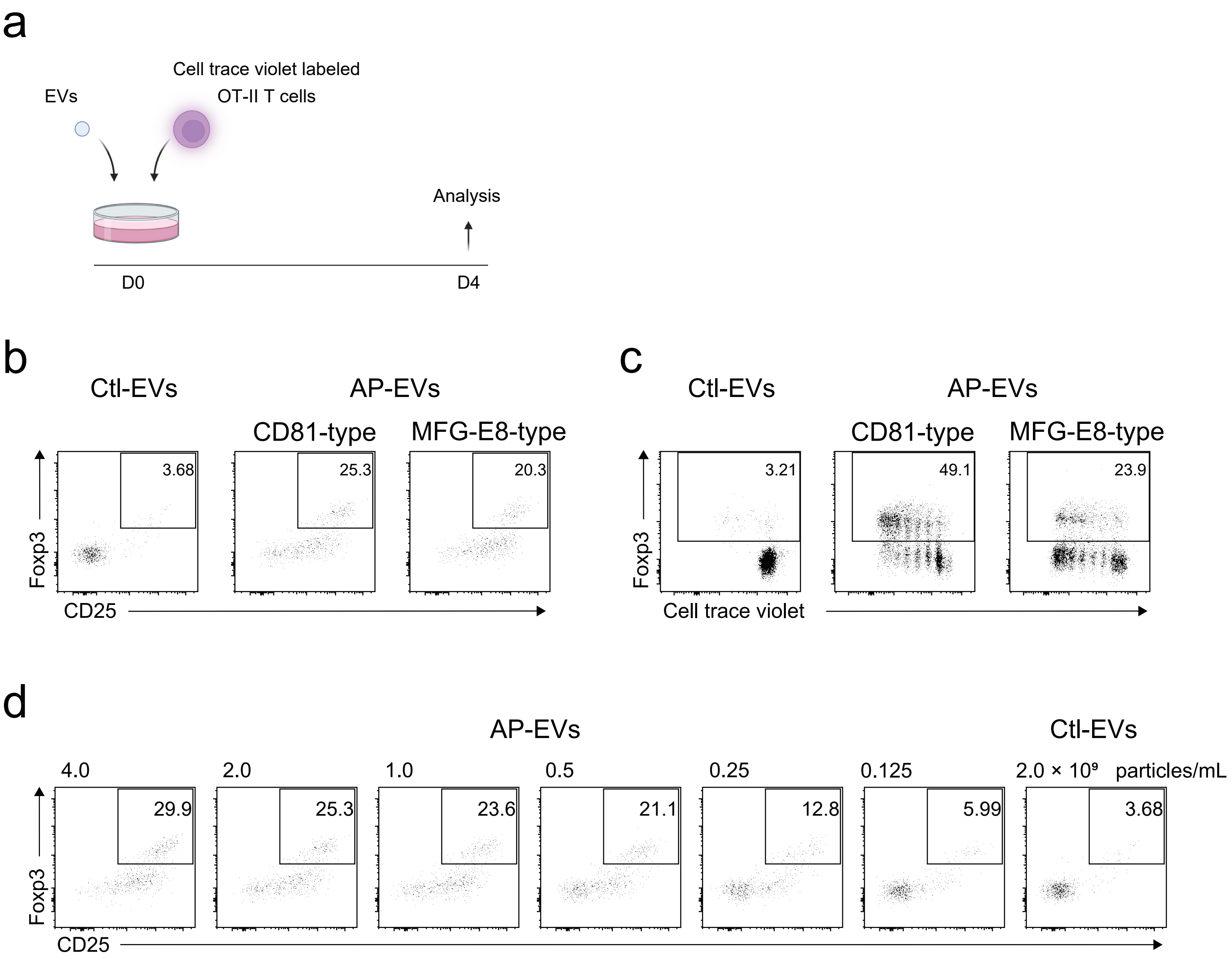

Supplementary Figure 3. Imai S, et al.

a

MOG-peptide-I-Aβ-CD81-IL-2 amino acid sequence

|← Signal peptide →|← MOG-peptide →|← Linker →|← I-Aβ

MALQIPSLLLSAAVVVLMVLSSPGTEGVGWYRSPFSRVVHLGGGGGGTSGGGSGGSGDSERHFVYQFMGECYFTNGTQRIRYVTR
YIYNREEYVRYDSDVGEHRAVTELGRPDAEYWNSQPEILERTRAELDTVCRHNYEGPETHTSLRRLEQPNVVISLSRTEALNHHNTL
VCSVTDFYPAKIKVRWFRNGQEETVGVSSTQLIRNGDWTFQVLVMLEMTPRRGEVYTCHVEHPSLKSPITVEWRAQSESAWSKMLS

I-Aβ →|← CD81

GIGGCVLGVIFLGLGLFIRHRSQKGPRGPPPAGLLQMGVEGCTKCIKYLLFVFNFVFWLAGGVILGVALWLRHDPQTTSLLYLELGNK
PAPNTFYVGIYILIAVGAVMMFVGFLGCYGAIQESQCLLGTFFTCLVILFACEVAAGIWGFVNKDQIAKDVKQFYDQALQQAVMDDDA

CD81 →|← Linker →|← IL-2

NNAKAVVKTFHETLNCCGSNALTTLTTTILRNSLCPSGGGGSAPTSSSTSSSTAEAQQQQQQQQQQQHLEQLLMDLQELLSRME
NYRNLKLPRMLTFKFYLPKQATELKDLQCLEDELGPLRHVLDLTQSKSFQLEDAENFISNIRVTVVKLKGSDNTFECQFDDESATVVD

IL-2 →|← Linker →|← CD81 →|

FLRRWIAFCQSIISTSPQGGGGSGGNILTPLLQQDCHQKIDELFSGKLYLIGIAAIVVAVIMIFEMILSMVLCCGIRNSSVY

b

MOG-peptide-I-Aβ-leucine zipper-MFG-E8 amino acid sequence

|← Signal peptide →|← MOG-peptide →|← Linker →|← I-Aβ

MALQIPSLLLSAAVVVLMVLSSPGTEGVGWYRSPFSRVVHLGGGGGGTSGGGSGGSGDSERHFVYQFMGECYFTNGTQRIRYVTR
YIYNREEYVRYDSDVGEHRAVTELGRPDAEYWNSQPEILERTRAELDTVCRHNYEGPETHTSLRRLEQPNVVISLSRTEALNHHNTLV

I-Aβ →|← Linker

CSVTDFYPAKIKVRWFRNGQEETVGVSSTQLIRNGDWTFQVLVMLEMTPRRGEVYTCHVEHPSLKSPITVEWRAQSESAWSKGGG

Linker →|← Leucine zipper →|← Linker →|← MFG-E8

GSLEIEAAFLERENTALETRVAELRQRVQRLRNRVSQYRTRYGPLGGGKGGGGSASGDFCDSSLCLNGGTCLTGQDNDIYCLCPE
GFTGLVCNETERGPCSPNPCYNDAKCLVTLDTQRGEIFTEYICQCPVGYSGIHCETETNYYNLDGEYMFTTAVPNTAVPTPAPTPDL
SNNLASRCSTQLGMEGGAIADSQISASSVYMGFMGLQRWGPELARLYRTGIVNAWTASNYDSKPWIQVNLLRKMRVSGVMTQGAS
RAGRAEYLKTFKVAYSLDGRKFEFIQDESGGDKEFLGNLDNNSLKVNMFNPTLEAQYIKLYPVSCHRGCTLRFELLGCELHGCSEPL
GLKNNTIPDSQMSASSSYKTWNLRAFGWYPHLGRLDNQGKINAWTAQSNSAKEWLQVDLGTQRQVTGIITQGARDFGHIQYVASYK

MFG-E8 →|

VAHSDDGVQWTVYEEQGSSKVFQGNLDNNSHKKNIFEKPFMARYVRVLPVSWHNRITLRLELLGC

c **d** **e** **f**

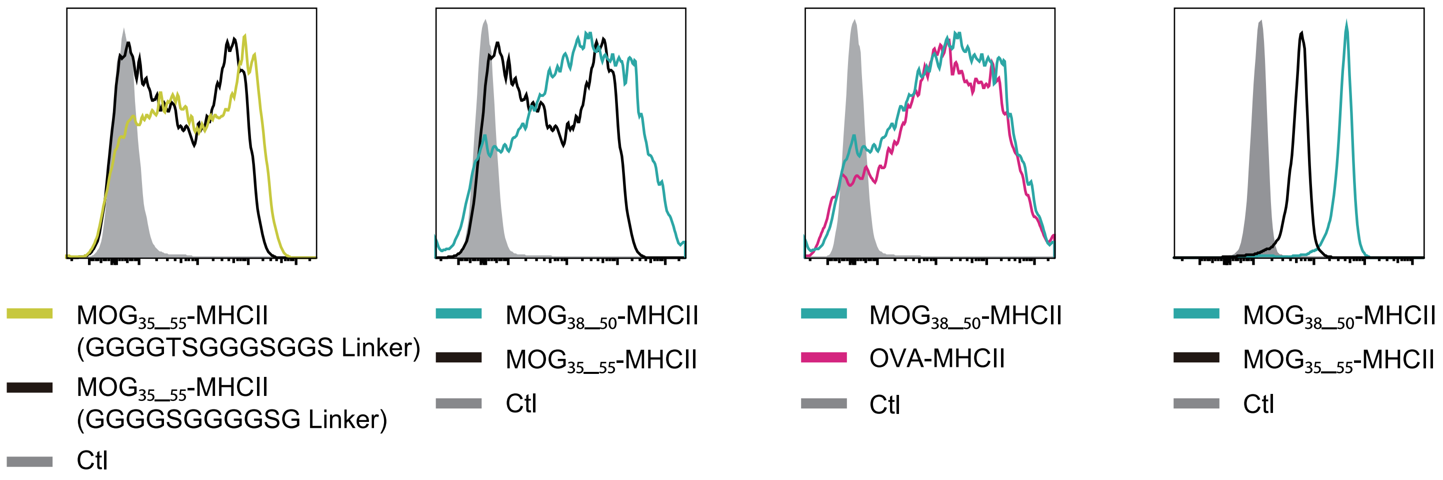

Supplementary Figure 4. Imai S, et al.

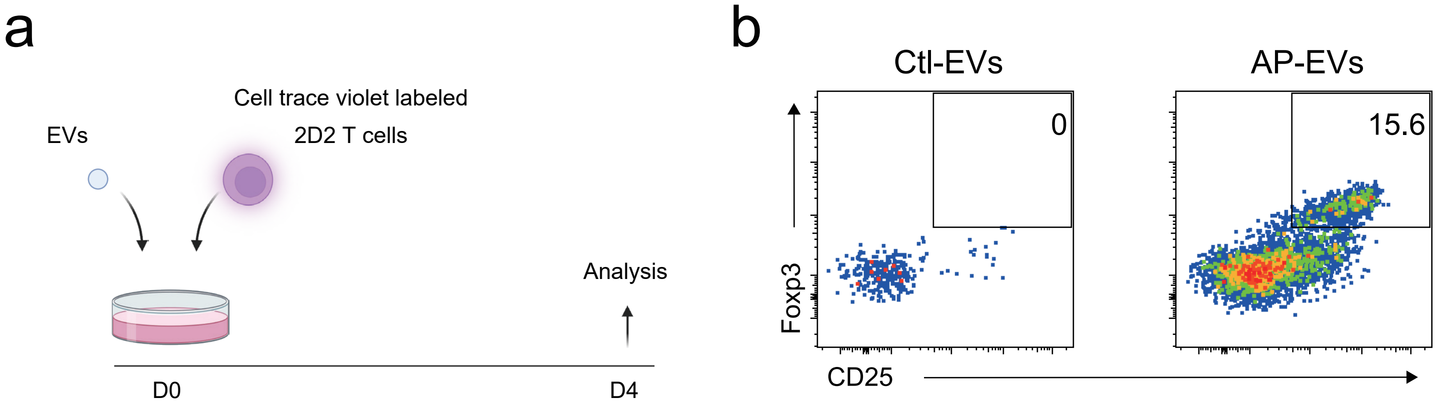

Supplementary Figure 5. Imai S, et al.

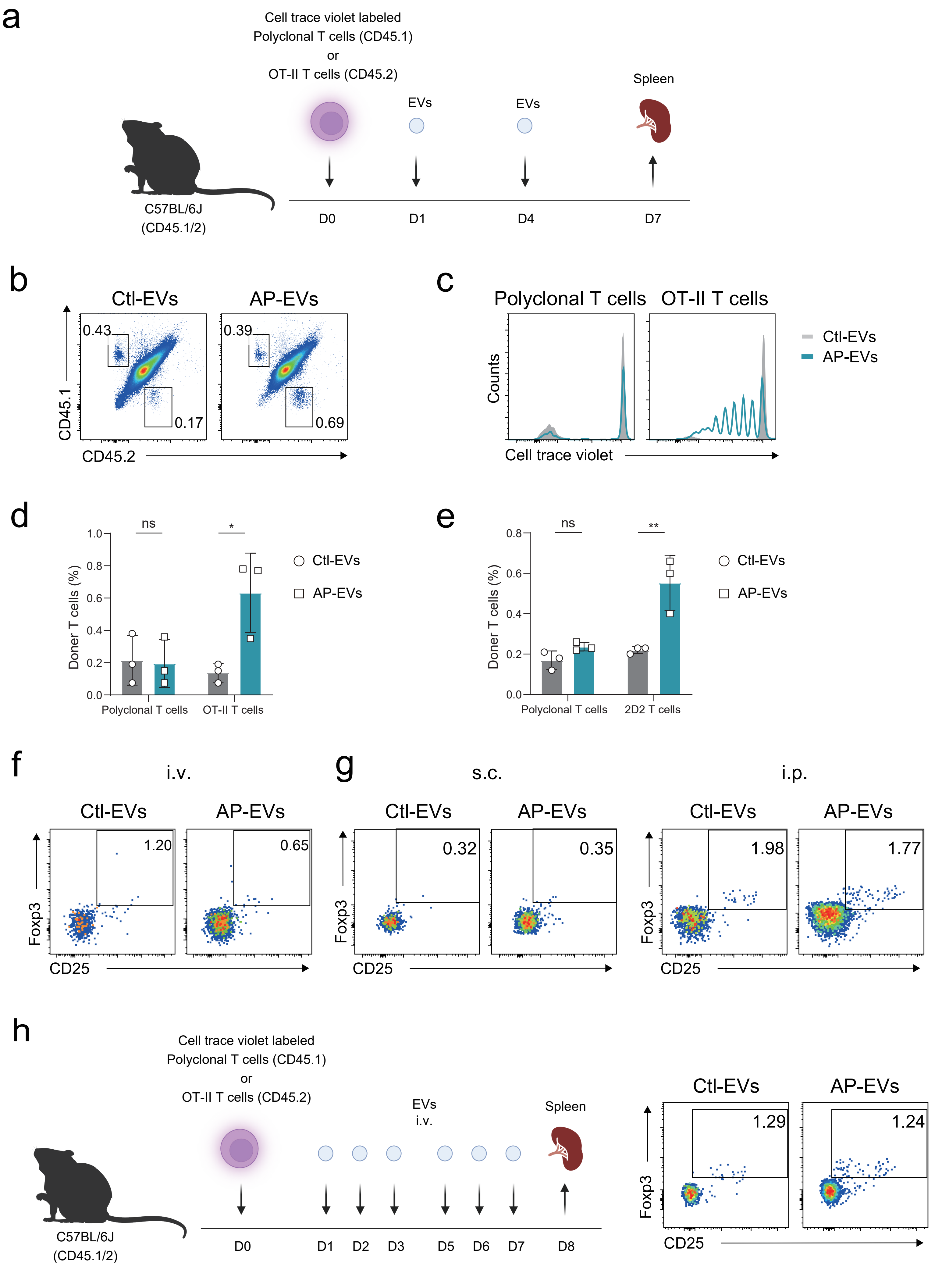

Supplementary Figure 6. Imai S, et al.

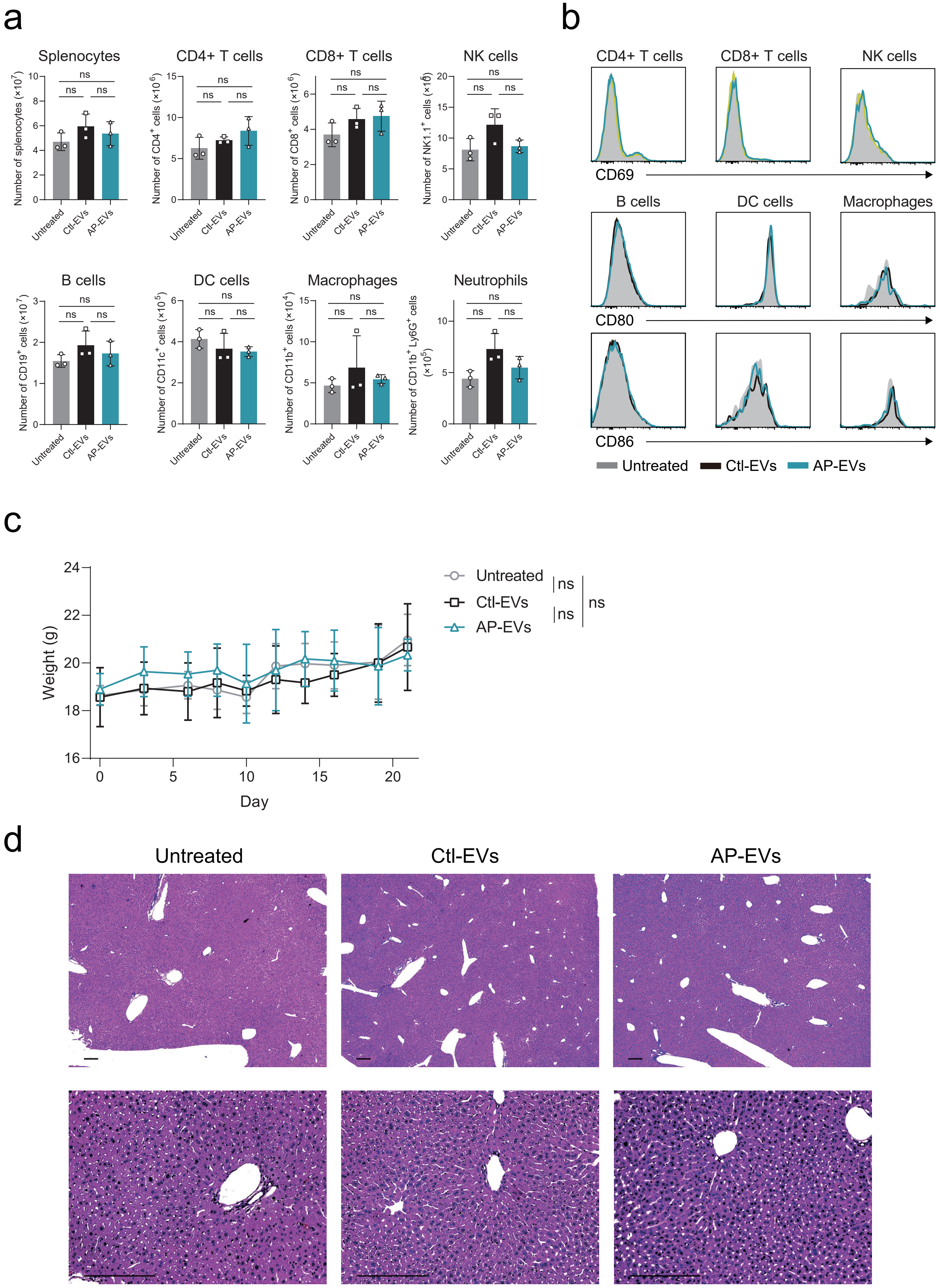

Supplementary Figure 7. Imai S, et al.

Figure. S1. Amino acid sequences of fusion proteins.

Amino acid sequences of the following constructs are shown: (a) I-Aα, (b) OVA peptide–I-Aβ–CD81–IL-2, (c) TGF-β–CD8–CD81, (d) I-Aα fused to a leucine zipper, (e) OVA peptide–I-Aβ–leucine zipper–MFG-E8, (f) TGF-β–MFG-E8–IL-2.

Figure. S2. Colocalization of MFG-E8 fusion proteins in EVs.

(a) The expression of human β-actin, human CD81, and human CD9 in HEK293T cells and EVs was analyzed by Western blotting. (b, c) HEK293T cells were co-transfected with plasmids encoding RFP–MFG-E8 and GFP–MFG-E8 fusion proteins. EVs were isolated by ultracentrifugation and analyzed by confocal microscopy. Colocalization of RFP^+ and GFP^+ fluorescent puncta were quantified. Scale bar indicates 4 μm. (d) Size distribution of CD81-type AP-EVs and control EVs measured by AFM.

Figure. S3. Induction of OVA-specific Treg differentiation by AP-EVs *in vitro*.

(a) A total of 2.0×10^5 CTV-labeled OT-II T cells were co-cultured with AP-EVs or control EVs for 4 days at a concentration of 2.0×10^9 particles/mL. (b) Treg differentiation was assessed by flow cytometry based on CD25 and Foxp3 expression. (c) On day 7 of culture, Foxp3 expression and CTV dilution were analyzed to evaluate Treg induction and proliferation. (d) OT-II T cells were cultured with CD81-type AP-EVs at concentrations ranging from 4.0×10^9 particles/mL to 0.125×10^9 particles/mL, and CD25 and Foxp3 expression was analyzed on day 4.

Figure. S4. Optimization of MOGp–MHCII constructs.

(a, b) Amino acid sequences of MOG peptide-I-Aβ-CD81-IL-2 and MOG peptide-I-Aβ-leucine zipper-MFG-E8 fusion constructs. (c) Comparison of MOG_{35-55}-MHCII constructs fused via different linker sequences (GGGGSGGGGSG or GGGGTSGGGSGGS) in HEK293T cells; surface expression of MHCII was evaluated one day after transfection. (d) Surface expression levels of MHCII molecules loaded with MOG_{38-50} or MOG_{35-55} peptides using the GGGTSGGGSGGS linker were compared. (e) Surface expression levels of MOG_{38-50}–MHCII and OVAp–MHCII on HEK293T cells were analyzed. (f) Surface expression of MHCII loaded with MOG_{38-50} or MOG_{35-55} peptides on EVs was evaluated by flow cytometry.

Figure. S5. Induction of MOG-specific Treg differentiation by AP-EVs *in vitro*.

(a, b) 2.0×10^5 CTV-labeled 2D2 T cells were co-cultured with AP-EVs or control EVs for 4 days. Treg differentiation by CD25 and Foxp3 expression were assessed by flow cytometry.

Figure. S6. *In vivo* induction of antigen-specific Tregs by AP-EVs in adoptive transfer models

(a) Schematic of the adoptive T cell transfer and EV treatment protocol. $CD45.2^+$ T cells from OT-II or 2D2 transgenic mice were mixed at a 1:1 ratio with $CD45.1^+$ T cells isolated from wild-type C57BL/6 mice. Total of 2.0×10^6 T cells were intravenously injected into CD45.1/2 congenic recipient mice. Mice received 5.0×10^{10} particles of either AP-EVs or control EVs on days 1 and 4 post-transfer.

(b–e) Donor-derived cell expansion was assessed by identifying $CD45.1^+$ and $CD45.2^+$ single-positive populations in the spleen 3 days after the final EV administration (n=3). (f) Treg differentiation of OT-II–derived $CD4^+$ T cells were evaluated by measuring Foxp3 and CD25 expression (n=3). (g) Mice received 5.0×10^{10} particles of either AP-EVs or control EVs on days 1 and 4 post-transfer via intraperitoneal or dorsal subcutaneous injection. (h) Mice were treated with the same dose of EVs once daily for six consecutive days following T cell transfer, administered through the intravenous injection. On day 8, spleens were harvested, and single-cell suspensions were prepared and analyzed by flow cytometry. All statistical analyses were performed with GraphPad Prism version 8.0. *P ≤ 0.05, **P ≤ 0.01, ***P ≤ 0.001, ****P ≤ 0.0001.

Figure. S7. *In vivo* immunogenicity and safety assessment of HEK293T-derived EVs

(a, b) Mice were administered 5.0×10^{10} particles of AP-EVs or control EVs. At 24 h post-administration, spleens were harvested, and total splenocyte counts as well as activation markers of antigen-presenting cells and lymphocytes were analyzed by flow cytometry (n=3). (c) Mice received 5.0×10^{10} particles of AP-EVs or control EVs every other day for a total of three injections. Body weight was recorded every other day or every two days (n=3). (d) Two weeks after the final injection, the liver was fixed and subjected to H&E staining. Scale bar indicates 200 μm. All histological images were acquired using the BZ-X series all-in-one fluorescence microscope. All statistical analyses were performed with GraphPad Prism version 8.0. *P ≤ 0.05, **P ≤ 0.01, ***P ≤ 0.001, ****P ≤ 0.0001.
